# Gustatory Rhinorrhea: A Rare Presentation of Parkinson’s Disease

**DOI:** 10.7759/cureus.3224

**Published:** 2018-08-29

**Authors:** Ujala Zubair, Osama Salam, Zarafshan Zubair

**Affiliations:** 1 Medicine, Dow University of Health Sciences (DUHS), Karachi , PAK; 2 Internal Medicine, Dow University of Health Sciences (DUHS), Karachi, PAK; 3 Medical Student, Dow University of Health Sciences (DUHS), Karachi, PAK

**Keywords:** gustatory rhinorrhea, parkinson's disease, rare presentations

## Abstract

Non-motor symptoms appear earlier than the motor symptoms of Parkinson’s disease. Gustatory rhinorrhea is a rare presentation of Parkinson’s disease. We report a case of 70-year-old male who presented to the outpatient department* *(OPD) with watery secretions on thought or site of food. Symptomatic treatment was advised along with many investigations for the cause. Twenty-two months later the patient developed fine tremors of hands which were evident of Parkinson’s disease.

## Introduction

Parkinson’s disease is a neurodegenerative disorder affecting the autonomic, limbic and somatomotor system and later involving the dopaminergic neurons of mid-brain and substantia nigra. The four most common pathways suffering damage in Parkinson's disease include the mesocortical, mesolimbic, tubero-infundibular and nigrostriatal pathways. These pathways are also involved in several non-motor functions [1,2].

Non-motor features of Parkinson's disease include hypotension, urinary incontinence, erectile dysfunction, anosmia, constipation, depression, sleep disorders and fatigue. These symptoms are rarely used for the diagnosis of Parkinson disease at an early stage [3].

Few studies have suggested that Parkinson disease pathology begins from extra-nigral structures such as olfactory bulb, brain stem nuclei and sympathetic plexus usually gastric myenteric plexus rather than substantia nigra pars compacta [1].

Some researchers have recommended that sympathetic denervation of the nasal mucosa is found in Parkinson’s disease patients which is followed by unopposed parasympathetic stimulation which explains dribbling of watery secretions. Sympathetic denervation followed by unopposed parasympathetic denervation is also found in heart among patients with Parkinson’s disease [4,5].

## Case presentation

We report a case of a 70-year-old male patient who presented to the outpatient department (OPD) with chief complain of dribbling of watery secretions from nasal mucosa on the thought of food or sight of food. Each episode of dribbling comprised of secretion of 50–100 ml of watery fluid. Past medical history was significant for hypertension for three years and type 2 diabetes mellitus for five years. There was no history of any neuro-degenerative disease in the family. Furthermore, there was a history of constipation for three months along with the presence of mucus in stool. Colonoscopy was performed which showed no significant pathology. There was no evidence of dementia or other psychiatric disturbances. Mild sleep abnormalities were present. Forward flex posture was present along with broad-based gait. A mask-like face was not present.

A provisional diagnosis of gustatory rhinorrhea was made and the patient was advised of anti-cholinergic medications and anti-histamine drugs. There was little to no benefit with these medications. The patient was further prescribed with nasal corticosteroid sprays, mucolytic medications, and nasal irrigations but these prescriptions only provided mild symptomatic improvement.

Twenty-two months later, the patient presented again in the OPD and had developed fine tremors in fingers and hands. These tremors were absent in head and lower limbs. Tremors were only evident at rest while absent on activity. Further examination revealed an altered sense of smell which was un-noticed by the patient. The patient was diagnosed with Parkinson’s disease. Dopamine-based therapy was commenced which resulted in improvement of rhinorrhea as well as motor symptoms.

## Discussion

In our patient, early diagnosis of Parkinson’s disease would have prevented the delay in commencement of neuro-protective strategies. There is a 2% chance of an individual over the age of 50 years to develop Parkinson’s disease. If family history is positive for Parkinson’s disease, the chance doubles to 4% [6]. There can be difficulties in diagnosis of Parkinson’s disease. Some common differentials can be Alzheimer’s disease, Lewy body dementia, and essential tremor. Around 25% cases are clinically misdiagnosed as Parkinson’s disease [7]. The response to dopamine therapy is the only confirmatory sign for correct diagnosis. However, some diagnostic studies have been used to diagnose and treat Parkinson’s disease at an early stage. These include genetic testing, neuroimaging, autonomic function testing studies and dopamine challenge testing [7]. Some recent studies have identified the role of transcranial sonography and 123I-B-SPECT (Single-photon emission computed tomography) in diagnosing Parkinson’s disease at the earliest possible stage [3].

Neuronal loss involving nigrostriatal neurons progresses up to 40% before the initial appearance of motor signs. In contrast to which non-motor symptoms appear early and can provide clues to early diagnosis [8,9]. The appearance of motor signs is further delayed by the compensatory mechanisms which delay the appearance of motor signs by counter-acting the striatal neuronal loss [10,11].

Chou et al. have concluded in their study that among Parkinson’s disease patients, rhinorrhea due to any cause is not related to the severity of nigrostriatal dysfunction [12]. The correlation among rhinorrhea, autonomic dysfunction, and sympathetic denervation needs further research which could help in understanding the underlying pathophysiologic mechanisms.

## Conclusions

Some motor tests can be important in early screening for Parkinson’s disease. In case of positive screening results, further imaging studies such as DAT imaging (a dopamine transporter single photon emission computerized tomography imaging technique) can be carried out for final diagnosis. Rare presentations should be kept in mind while examining high risk individuals.

## References

[1] Braak H, Ghebremedhin E, Rüb U, Bratzke H, Del Tredici K (2004). Stages in the development of Parkinson’s disease-related pathology. Cell Tissue Res.

[2] Braak H, Del Tredici K, Rüb U, de Vos RA, Jansen Steur EN, Braak E (2003). Staging of brain pathology related to sporadic Parkinson’s disease. Neurobiol Aging.

[3] Schrag A, Horsfall L, Walters K, Noyce A, Petersen I (2015). Prediagnostic presentations of Parkinson's disease in primary care: a case-control study. Lancet Neurol.

[4] Friedman JH, Amick MM, Chou KL (2008). Rhinorrhea and olfaction in Parkinson disease. Neurology.

[5] Fujishiro H, Frigerio R, Burnett M (2008). Cardiac sympathetic denervation correlates with clinical and pathologic stages of Parkinson's disease. Mov Disord.

[6] Elbaz A, Bower JH, Maraganore DM (2002). Risk tables for parkinsonism and Parkinson's disease. J Clin Epidemiol.

[7] Tolosa E, Wenning G, Poewe W (2006). The diagnosis of Parkinson's disease. Lancet Neurol.

[8] Greffard S, Verny M, Bonnet AM (2006). Motor score of the Unified Parkinson Disease Rating Scale as a good predictor of Lewy body-associated neuronal loss in the substantia nigra. Arch Neurol.

[9] Fearnley JM, Lees AJ (1991). Ageing and Parkinson's disease: substantia nigra regional selectivity. Brain.

[10] Bezard E, Gross CE, Brotchie JM (2003). Presymptomatic compensation in Parkinson's disease is not dopamine-mediated. Trends Neurosci.

[11] van Nuenen BF, van Eimeren T, van der Vegt JP, Buhmann C, Klein C, Bloem BR, Siebner HR (2009). Mapping preclinical compensation in Parkinson's disease: an imaging genomics approach. Mov Disord.

[12] Chou KL, Koeppe RA, Bohnen NI (2011). Rhinorrhea: a common nondopaminergic feature of Parkinson's disease. Mov Disord.

